# Diversity trends in bread wheat in Italy during the 20th century assessed by traditional and multivariate approaches

**DOI:** 10.1038/srep08574

**Published:** 2015-02-25

**Authors:** Leonardo Ormoli, Corrado Costa, Stefano Negri, Maurizio Perenzin, Patrizia Vaccino

**Affiliations:** 1 Consiglio per la ricerca in agricoltura e l'analisi dell'economia agraria - Unità di ricerca per la selezione dei cereali e la valorizzazione delle varietà vegetali (CRA-SCV) via R. Forlani 3, 26866 Sant'Angelo Lodigiano (LO) – Italy; 2 Consiglio per la ricerca in agricoltura e l'analisi dell'economia agraria - Unità di ricerca per l'ingegneria agraria (CRA-ING) via della Pascolare, 16, 00015 Monterotondo Scalo (RM) – Italy

## Abstract

A collection of 157 *Triticum aestivum* accessions, representative of wheat breeding in Italy during the 20^th^ century, was assembled to describe the evolutionary trends of cultivated varieties throughout this period. The lines were cultivated in Italy, in two locations, over two growing seasons, and evaluated for several agronomical, morphological and qualitative traits. Analyses were conducted using the most common univariate approach on individual plant traits coupled with a correspondance multivariate approach. ANOVA showed a clear trend from old to new varieties, leading towards earliness, plant height reduction and denser spikes with smaller seeds. The average protein content gradually decreased over time; however this trend did not affect bread-making quality, because it was counterbalanced by a gradual increase of SDS sedimentation volume, achieved by the incorporation of favourable alleles into recent cultivars. Correspondence analysis allowed an overall view of the breeding activity. A clear-cut separation was observed between ancient lines and all the others, matched with a two-step gradient, the first, corresponding roughly to the period 1920–1940, which can be ascribed mostly to genetics, the second, from the 40s onward, which can be ascribed also to the farming practice innovations, such as improvement of mechanical devices and optimised use of fertilizers.

Germplasm collections represent valuable genetic resources for breeding programmes aimed at delivering new superior cultivars that are better suited to the technological and nutritional needs of the market. Traditionally, germplasm collections are screened for phenotypic traits and the lines presenting the desired phenotype are then crossed with an elite cultivar, to incorporate the desired trait into the elite genetic background. In contrast to such a “top-down” approach, an alternative “bottom-up” strategy was recently developed, which starts with the analysis at the genomic sequence level, and then works backwards up to the phenotype by means of linkage disequilibrium through genome wide association scan (GWAS)^1^. When this type of analysis is performed across a highly diverse set of accessions, which contain many more historical recombination events compared to the bi-parental populations commonly used in linkage analysis, a much higher mapping resolution is achieved. Recently, GWAS has been carried out successfully in wheat for the identification of Quantitative Trait Loci (QTLs) linked to yield and agronomic traits^2^,^3^,^4^ and resistance to pathogens^5^,^6^,^7^. The huge size of many germplasm collections, however, often hinders their exploitation; hence, the need to focus on core collections, i.e. representative manageable samples better suited to perform the initial assessments, before re-exploring broader ranging materials^8^.

Given all the previous assumptions, within the *Triticum aestivum* collection available at CRA-SCV, including more than 4000 accessions, a sub-collection was identified and a GWAS project was started to identify possible linkages between molecular markers and relevant breeding traits. The accessions, selected following a temporal principle (year of constitution or diffusion) integrated with agronomic and qualitative information, summarize the process of wheat breeding in Italy during the last hundred years.

Historical series of genotypes have been used in different countries to assess the genetic gains achieved after the onset of modern wheat breeding^9^,^10^,^11^,^12^,^13^,^14^,^15^,^16^. The prevailing factor in all the environments was the breeding success in increasing grain yield potential, largely attributed to changes in biomass partitioning, *i. e.* harvest index. The increase of harvest index was in turn the consequence of the decrease of plant height and of higher grain number per square meters of modern cultivars, while changes in total biomass production and grain weight did not show a clear contribution to yield gains worldwide^11^,^17^,^18^,^19^,^20^. The majority of these studies used the standard univariate approach on individual plant traits, obtaining precise but individual snapshots of the overall changes fostered by the breeding process. Multivariate ordination methods such as Principal Components Analysis and Correspondance Analysis, on the other hand, help in interpreting the genetic control of multiple traits^21^. Correspondence analysis also permits to analyze together qualitative and categorized quantitative variables, allowing the simultaneous representation of variables and observations^22^,^23^. Aim of the present work was the accurate phenotyping of a wide germplasm collection for several agronomic, morphological and qualitative traits to assess, by means of univariate and multivariate analyses, the trends of bread wheat breeding in Italy over the 20^th^ century and to survey the shift of key traits.

## Results

### Morpho-physiological characterization

**Figure 1:**
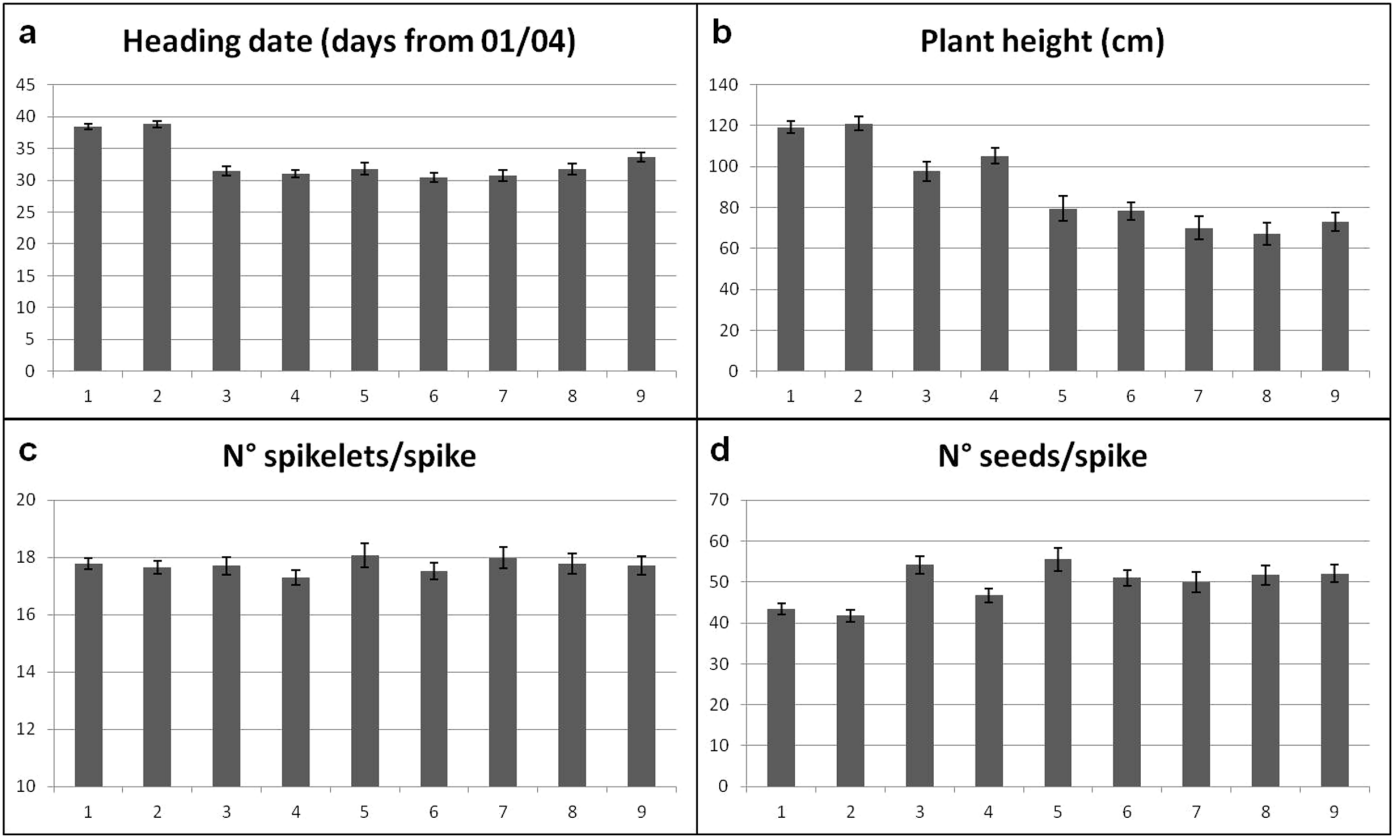
Average heading date (a), plant height (b), number of spikelets per spike (c) and number of seeds per spike (d) of the nine cultivar groups across four environments. Error bars indicate standard error; see Supplementary Table S1 for groups composition.

The ANOVA results for the recorded morpho-physiological and qualitative traits are summarised in Table 1. Significant differences due to both sources of variations were observed for heading date, plant height and seeds per spike, while spikelets per spike was influenced only by line within group. The interactions, tested using the pooled error of the check cultivar Salmone, were always significant, except for lines within group × environment of the trait spikelets/spike. The average heading date values for each of the nine groups identified in the core collection are reported in Fig. 1(a). A remarkable difference was evident between the landraces and their selections (Groups 1 and 2) *versus* all further groups, resulting in a gain of about one week in earliness for the cultivars belonging to Groups 3–9. Moreover, Groups 1 and 2 showed the highest within-group variation: both the latest (Inallettabile 961, 45 days) and the earliest (Chianti, 27 days) accession belonged to the first two groups.

A strong decrease in plant height was observed from the oldest to the more recent accessions (Fig. 1(b)) with a reduction of more than 80 cm from the tallest (Inallettabile 8, Group 2: 137 cm) to the shortest (Golia, Group 8: 54 cm) line. Lodging also showed a clear trend toward reduced susceptibility in the more recent accessions (results not shown): the highest lodging (70% of plants) was observed for Cologna Lunga (Group 1, 134 cm tall). No lodging was observed in Groups from 5 to 9.

The growth habit of young plants (results not shown) was generally erect or semi-erect; only Solina (Group 1), Inallettabile 961 and Inallettabile Todaro (Group 2) had a prostrate growth habit.

The number of spikelets per spike, as already stated, did not change significantly among groups (Fig. 1(c)) and showed a very narrow range of variation, from 17.3 (Group 4) to 18.1 (Group 5). On the contrary, the number of seeds per spike increased from the most ancient lines up to 56 in Group 5, followed by a slow decrease and a stabilization at 52 in the most recent varieties (Fig. 1(d)).

As expected, spike shape, density, awnedness and spike and seed colour largely depend on the genotype and did not show variations between locations and years (not shown). The most frequently observed spike shapes were oblong and fusiform. In general, the most ancient lines had lower spike density, ranging from lax to very lax. No specific trend throughout the groups could be detected for awnedness and glume colour. As far as seed characters are concerned, the prevalence of red colour (present in 90% of the lines), and the tendency towards reduced seed size from the oldest lines to the more recent ones, were observed.

### Grain quality traits

**Figure 2:**
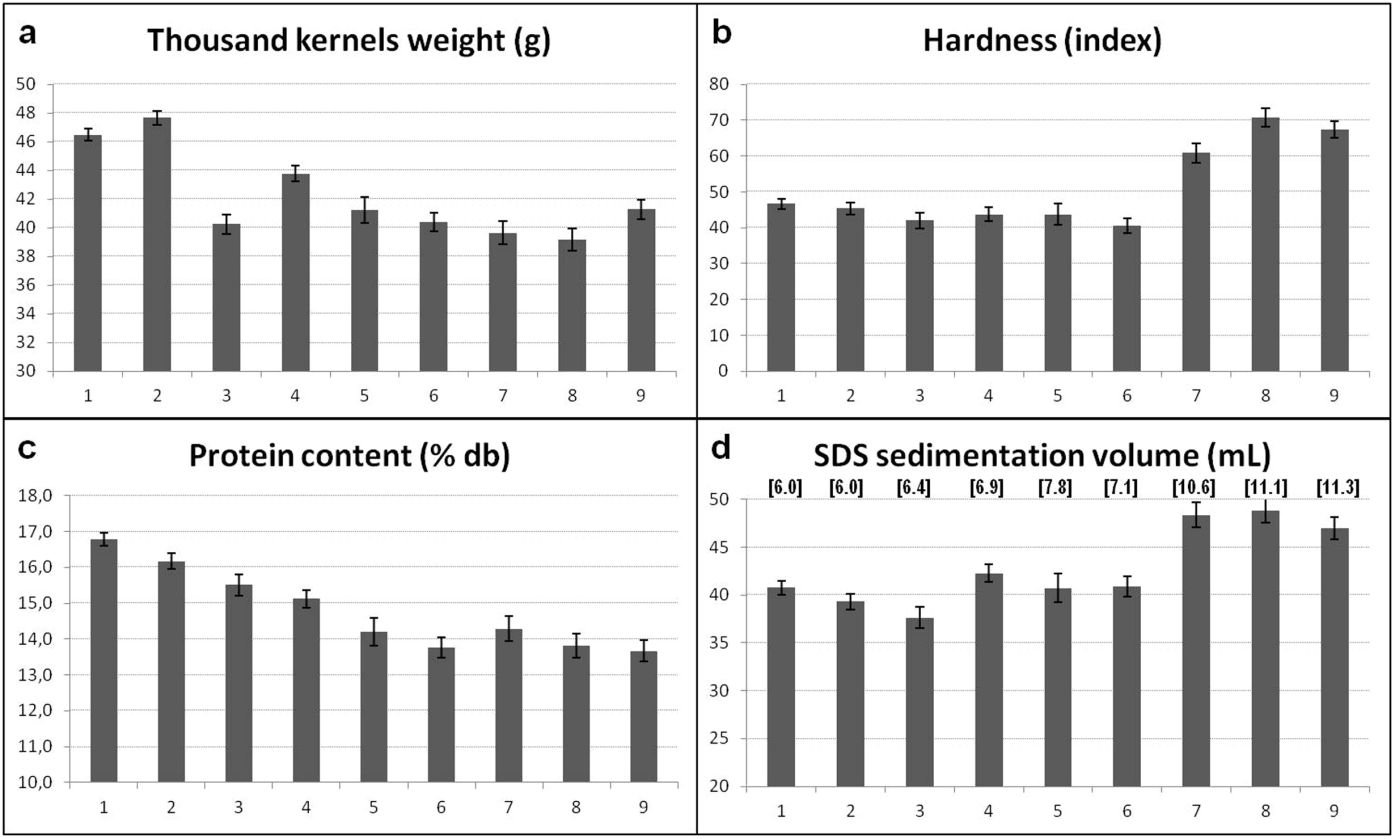
Average thousand kernels weight (a), hardness (b), protein content (c) and SDS sedimentation volume (d) of the nine cultivar groups across four environments. In (d) the average quality scores of the groups, deriving from the HMW-GS composition, are reported in square brackets. Error bars indicate standard error; see Supplementary Table S1 for groups composition.

Variations due to group and lines within group significantly affected all the traits; the interactions were always significant (Table 1). TKW decreased over time (Fig. 2(a)): the highest average values were registered in Group 1 (46.2 g) and Group 2 (47.7 g), comprising the more ancient lines, and the lowest in Group 8 (39.2 g), which includes the varieties released in the ‘90s of the past century. On the other hand, a strong hardness increment starting from Group 7 was observed (Fig. 2(b)). The average protein content (Fig. 2(c)) gradually decreased from group 1 (16.8%) to group 9 (13.7%). However, this trend did not affect bread-making quality, because it was counterbalanced by a gradual increase of SDS sedimentation volume (SSV), a good indicator of gluten quality: the highest values of SSV were in fact found in groups 7, 8 and 9 (Fig. 2(d)). The results were confirmed when scaling SSV to protein content: the lines belonging to groups 7, 8 and 9 had the highest SSSV values (see Supplementary Fig. S1).

### Multivariate analysis

**Figure 3:**
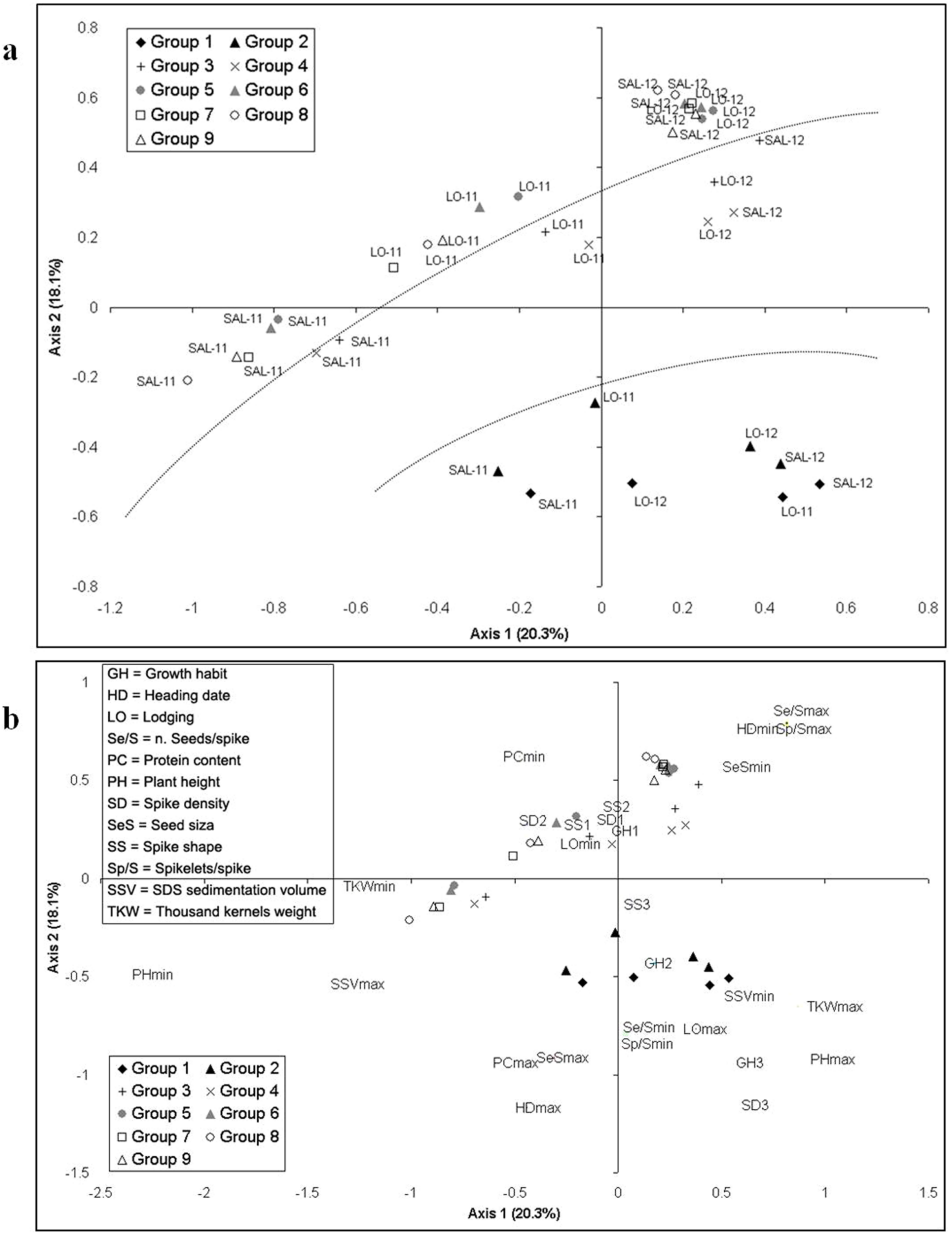
Correspondence analysis: factorial plane representation for the 36 cases represented by the product of 2 sites (SAL and LO) per 2 years (2011 and 2012) per 9 groups. The dotted lines in (a) do not have any statistical significance, but were drawn to help in highlighting some trends. In (b) the extreme values of the most representative variables are plotted. Quantitative variables are expressed as the minimum (min) and maximum (max) group variable. Qualitative variables are represented with numbers associated to the variable level.

The representation of the factorial plane, comprising 36 cases represented by the product of 2 sites (SAL and LO) per 2 years (2011 and 2012) for 9 groups, is reported in Figure 3. This plane represents 38.4% of the total information embedded in the original data matrix.

The 2011 observations were positioned in the left side of Axis 1 (all negative scores, apart one from Lodi). Moreover, among the 2011 observations, those from SAL generally showed lower scores than the ones from LO on the first axis. Such representation allows the identification of a clear effect of the year of cultivation and, for 2011, of the site. In particular, the 2010–11 growing season was characterised by high rainfall during the trimester october-december, which strongly influenced the sowing operations, delayed by about one month at SAL in comparison to LO, producing differences in the plant growth cycle, with consequences on the heading date and on the morphology of the plants. A clear-cut separation is evident on axis 2 between the more ancient groups 1 and 2, represented by the landraces and their selections, which are positioned in the lower-right side of the scatter plot, and all the other groups. A less clear gradient can be observed, going from groups 1 and 2 to groups 3 and 4, to the remaining ones (Fig. 3(a), dotted lines).

In Figure 3(b) the factorial plane representation for the 36 cases depicted in Figure 3(a) is superimposed with the plotting of the extreme values of the most representative variables. As a consequence the observations and the more representative variables are displayed simultaneously on the factorial planes indicating the correspondence cases and the variables. Quantitative variables are expressed as the minimum (min) and maximum (max) group variable. Qualitative variables are represented with numbers associated to the variable level. The variables oriented on axis 1 (left-right and vice versa), therefore more affected (influenced) by the growing season than the others, were: plant height (PH), SDS sedimentation volume (SSV), and thousands kernels weight (TKW). The variables oriented approximately on axis 2 (from top to bottom and vice versa), and so more linked with the breeding period, are growth habit (GH), heading date (HD), lodging (LO), number of seeds/spike (Se/S), protein content (PC), spike density (SD), seed size (SsS), spikelets/spike (Sp/S).

### Seed storage proteins characterization

The subunit composition at *Glu-A*1, *Glu-B*1 and *Glu-D*1 (the three main loci coding for the HMW glutenins) of the accessions analysed is reported in Supplementary Table S2, together with the quality score deriving from HMW-GS composition^24^.

The analyses revealed a certain heterogeneity, particularly within the landraces (Group 1). The most heterogeneous line was Solina in which, out of the five seeds analyzed for each sample, two to three different subunit compositions at each locus could be observed. The heterogeneity of the landraces was expected, as already observed both in bread and durum wheat^25^,^26^: such lines in fact consist of a mix of different biotypes which evolved, often isolated, during long temporal frames.

As regards the allelic composition of the whole collection, three pattern were observed at the *Glu-A1* locus (N, 1, 2*), 14 at *Glu-B1* (6, 7, 7 + 8, 7 + 9, 6 + 8, 13 + 16, 13 + 19, 14 + 15, 17 + 18, 18*, 18 + 9, 20, 22, 26 + 27) and 5 at *Glu-D1* (2 + 12, 2 + 12*, 2 + 12**, 3 + 12, 5 + 10). The most frequent component at *Glu-A1* locus was subunit 1, with a 38,6% frequency, while the combinations 7 + 8 (31.7%) and 2 + 12 (78.2%) were the most common at *Glu-B1* and *Glu-D1* loci, respectively.

It is interesting to note, in the first groups, the prevalence of the subunit pair 2 + 12 at the *Glu-D1* locus, generally associated with lower breadmaking quality in comparison with its counterpart 5 + 10, which is abundant in the most recent cultivars (groups 8 and 9). The subunit pair 5 + 10 was observed, nearly always together with 2 + 12, in some lines belonging to Groups 1 and 2, was completely absent in Groups 3 to 5, and gradually increased in frequency from 6.2 to 71.4% from groups 6 to 9. In old accessions, some other poor-quality polypeptides coded by *Glu-B1* locus, such as subunits 20, 7, 18*, are frequent, while the good-quality combinations 7 + 9 and 17 + 18 were introduced more recently.

The quality score gradually increased from the value of 6.0, registered in Groups 1 and 2, to 11.3 (Group 9), in accordance to the qualitative data reported.

## Discussion

Plant height reduction and earlier plant heading were simultaneously achieved in the very first stages of wheat breeding in Italy, thanks to the pioneering activity of Nazareno Strampelli. Unaware of Mendel's laws of inheritance, he realized that significant improvements could not be obtained by intrapopulation selection (commonly used at the beginning of the 20^th^ century) and started a crossing program involving Italian and foreign varieties. The Japanese variety Akakomugi, in particular, was essential because of the strong linkage between the genes controlling short straw and earliness (*Rht*8*c* and *Ppd*-D1, respectively) carried by this line^27^. These two traits, once incorporated together in new varieties, likely improved yield potential in two ways: height reduction permitted to overcome lodging and earliness allowed to avoid late pathogen attacks and to escape the “stretta”, an anticipated termination of wheat life cycle due to high temperatures and water depletion in the soil. Plant height reduction was a worldwide trend in wheat breeding during the 20^th^ century: selection for shorter plants is reported for Argentina^28^, Australia^18^, the UK^29^, the United States^9^, France^20^, Canada^30^, Spain^16^, Scandinavia^15^,^31^. In Italy, the same trend was observed both in common^11^,^32^ and in durum wheat^33^,^34^.

The number of spikelets/spike did not change over time, while seeds/spike increased after the first crosses and then remained fairly constant (Figure 1d). Studies on the physiological basis of genetic gains generally showed that number of grains/m^2^, and above all number of grains/ear, are the main trait contributing to yield rise^10^,^11^,^16^,^28^,^35^,^36^. The improvement of the number of grains/ear is partly due to the introduction of dwarfing genes^37^. Since the Green revolution (1950–60), the use in breeding of the short-straw variety Norin 10 introgressed the *Rht-B1b* and *Rht-D1b* dwarfing genes in modern cultivars, leading also to spikes modification, resulting in more kernels/spike, but with little effect on the number of spikelets/spike^38^.

In our collection, a strong reduction of kernel weight from the landraces to the first cultivars released in the 1920s was observed, followed by a more gradual decrease during the ensuing decades; only the cultivars bred in the last 10 years showed slightly bigger seeds, likely because of the longer grain filling period associated to their slightly later heading time. In general, little change for grain weight in the twentieth century is reported^15^,^20^,^33^,^36^,^39^ although, in accordance with our results, many authors noticed that grain weight was reduced by genetic improvement^17^,^18^,^40^ and in only a few cases were increases in this trait reported^9^,^28^.

A strong influence of the processing industry on Italian breeding is evident from the 1970s, when the increase in breadmaking quality, evidenced by higher sedimentation volumes, was achieved. This improvement was obtained not by increasing the protein content, which, on the contrary, progressively diminished, but by selecting prolamin subunits conferring better quality to the gluten, after extensive genetic and biochemical studies demonstrated that differences in the number and type of HMW and LMW glutenin subunits and their interactions strongly affected breadmaking quality (for a comprehensive dissertation on gluten quality see Ref 41). Grain protein decrease over time is well documented both in bread^11^,^17^,^29^,^32^,^42^ and durum wheat^33^. According to Fufa *et al*., it is related to increased yield potential^42^: plant breeders, selecting for higher grain yield, indirectly chosed cultivars with lower protein content, probably because of a dilution effect of the proteins linked to the increased amount of carbohydrates^43^. A by-product of the selection for high breadmaking quality was the increase in kernel hardness, since wheat with harder texture produce flours with higher fermentation rates and dough water absorption, highly desirable traits in breadmaking^44^.

Quality improvement was mainly due to the introduction in Italian germplasm (from the 1970s) of the subunit pair 5 + 10 from Canadian, Russian and Australian varieties^45^. The presence of 5 + 10 in groups 1 and 2 was therefore unexpected, suggesting misclassification. Additionally, except for one case, the subunit pair 5 + 10 was always present together with its counterpart 2 + 12 in the lines belonging to these two groups, thus indicating heterogeneous samples; due to the nature of the material, we could not reject any accession. On the other hand, it can be speculated that such heterogeneity naturally arose in those populations but, due to the lack of detection tools (the electrophoretic methods were developed in the last fifty years) no selection for specific alleles was carried out on those materials.

The 20^th^ century breeding activity is summarised by the results of correspondence analysis. A clear-cut separation between ancient accessions and all the others is observed; a less evident gradient can be detected from groups 1 and 2 to groups 3 and 4, and then to all the remaining ones. The first gradient step, corresponding roughly to the period 1920–1940, can be ascribed to genetics: the strong modifications observed are consequences of thorough genome recombination deriving from the hybridization approach. The second step, from the 1940s onward, can be ascribed to genetics but also to the farming practice innovations, such as improvement of mechanical devices and optimised use of fertilizers^45^. Strong differences between ancient and modern lines are well-documented in both common and durum wheat^14^,^15^,^16^,^25^,^26^. Old accessions are important reservoirs of genetic variability and today their cultivation is increasing in Italy, supported by the widespread belief that they are rich in health-beneficial compounds; an opinion supported by the results of some research, which underscore significant differences between old and modern wheats for a range of phytochemicals^46^,^47^,^48^,^26^. Moreover, the idea is emerging that the increase of gluten-related diseases, such as “gluten sensitivity” could be related to the exposure to gluten macropolymers; these huge molecules are particularly abundant in modern varieties, which were purposely selected for the high quality of their flours, following the requirements of the bread making industry.

In conclusion, the selective pressure applied by Italian breeders in the XX century led to a deep change of bread wheat plant ideotype. Starting from the 1970s, the pressing requirements of the processing industry oriented the breeders' interest towards an increase in breadmaking quality. The broad range of variation for several plant and quality traits present in the Italian working collection highlights the valuable genetic potential of this genepool for advanced breeding programs.

## Methods

### Plant material and experimental conditions

A set of 157 bread wheat accessions, representative of wheat breeding in Italy from the beginning of the 20^th^century up to the present, was selected from the germplasm bank of CRA-SCV (see Supplementary Table S1). The accessions were arbitrarily classified into nine groups on the basis of their date of release or registration (varieties), or of cultivation (landraces). Group 1 consists of local landraces, group 2 includes selections within landraces, group 3 comprises the earliest cultivars obtained by Nazareno Strampelli by crossing Italian and foreign germplasm. Each of the remaining groups consists of cultivars released in the following (consecutive) 20-years period and having at least one of the parents belonging to the preceding group (Supplementary Table S1). In the first two groups, we included several accessions registered with the same name, but presenting morphological differences at a preliminary evaluation.

The accessions were cultivated in Italy, in two locations of the Northern Po valley, by two growing seasons, 2010–2011 and 2011–2012, following a maize crop. The locations were S. Angelo Lodigiano (SAL, silty sand soil, organic matter 27.8 g kg^−1^, N content 1.5 g kg^−1^, pH 7.1) and Lodi (LO; sandy silt soil, organic matter 16.6 g kg^−1^, N content 1.27 g kg^−1^, pH 6.2). The lines were hand-sown in single rows 1.0 m long and 0.40 m apart, following an unreplicated completely randomized layout with replicated check plots, represented by the cultivar Salmone repeated every 15 rows. The main cultural practices consisted in nitrogen application at tillering (40 kg N ha^−1^, as ammonium nitrate) and manual control of the weeds. The single rows were harvested by hand.

### Morpho-physiological characterization

Heading date (HD) was recorded as the number of days after April 1^st^ when about half of the culms showed emerging spikes; growth habit of young plants (GH) was scored as erect, semi-erect or prostrate; plant height (PH) was measured at maturity, as the average height (in cm) from the ground to the tip of the spike, excluding the awns; lodging (LO) was recorded on a 0–9 scale, where 0 = no lodging and 9 = all plants lodged. After harvest, on a random sample of five spikes per accession per location, spike shape (SS; 1: fusiform, 2: clavate, 3: oblong), colour (SC; 1: red to brown, 2: white/amber), density (SD; 1: lax, 2: intermediate, 3: very lax), and awnedness (AW; 1: none or <10 mm long awns, 2:10–40 mm long awns, 3: awns longer than 40 mm, 4: awns longer than spike), as well as seed colour (SeC; 1: red to brown, 2: white/amber) and size (SeS; 1: <7 mm, 2: 7–9 mm, 3: 10–12 mm; 4: > 12 mm) were registered, according to the descriptors suggested by IBPGR^49^. The number of spikelets per spike (Sp/S) and seeds per spike (Se/S) was also determined.

### Grain quality traits

Thousand-kernel weight (TKW) was determined on two 100-kernels samples. Eighty grams of each line were subsequently ground to wholemeal using a 1-mm-sieve Cyclotec mill (Foss Tecator AB, Höganäs, Sweden). Protein content (PC) (N × 5.7, dry weight, AACC 39–10^50^), and hardness (Ha) (AACC 39–70^50^) were determined by a NIR System Model 6500 (FOSS NIRSystems, Laurel, MD). The SDS sedimentation volume (SSV) was measured according to Preston *et al.*^51^. Specific SDS Sedimentation Volume (SSSV) was calculated as the ratio between SSV and PC. All the analyses were repeated twice (technical replicates) for each sample.

### Seed storage proteins characterization

Each accession was characterized for high molecular weight glutenin subunits (HMW-GS) composition, as described in Vaccino *et al*^52^. Five single caryopses and a bulk sample deriving from the grinding of about 100 seeds were analysed for each accession, in order to check the homogeneity of the samples. The identification of the different subunits was performed by comparing their electrophoretic mobilities to those of well-known bread wheat standards. The nomenclature proposed by Payne and Lawrence^53^ and Pogna *et al.*^24^ for HMW-GS was adopted.

### Data analysis

The data deriving from quantitative variables (HD, PH, Sp/S, Se/S, TKW, Ha, PC, SSV) were analysed by univariate analysis of variance (ANOVA). The combinations of two locations by two growing seasons were considered as variants of the random factor Environment. We adopted a mixed model ANOVA in which the fixed factors Groups (G) and Lines within Groups [L(G)] were tested using G × E and [L(G) × E], respectively, as the error terms. The check cultivar Salmone was used to estimate the experimental error. Its average mean square across the four environments was used, as pooled error, to test the significance of the G × E and [L(G) × E] interactions. The univariate analyses were carried out using SAS software (v9.4, SAS Institute Inc., Cary, NC).

To analyze together the variables deriving from qualitative variables (GH, LO, SS, SC, SD, AW, SeC, SeS) and quantitative variables, a multivariate approach consisting in correspondence analysis (CA) was adopted. For the analysis, the quantitative variables were converted into categories (groups) as follow:

HD: since April 1st, 8 groups (step: 5 days);

PH: group 1 < 50 cm, 6 groups (step: 15 cm), group 8 > 140;

Sp/S: group 1 < 14, 4 groups (step: 2), group 6 > 22;

Se/S: since 20, 7 groups (step: 9);

TKW: group 1 < 30, 4 groups (step: 10), group 6 > 70;

Ha: group 1 < 40 (soft), 40 < group < 70 (medium), group 3 > 70 (hard);

PC: group 1 < 12, 4 groups (step: 2), group 6 > 20;

SSV: group 1 < 20, 7 groups (step: 7), group 9 > 68.

The groups, considering 8 quantitative variables and 8 qualitative variables, are 84. As a consequence, the design matrix (Burt table), consists in the frequencies of 84 variables (columns) in 36 cases (rows), represented by the product of 2 sites per 2 years per 9 groups. The multivariate analysis was carried out using the PAST software^54^.

## Author Contributions

P.V. planned and coordinated the work, participated in the interpretation of the results and wrote the main manuscript text; L.O. performed the data collection; C.C. carried out the statistical analyses; M.P. participated in data analyses; all authors reviewed the manuscript.

## Supplementary Material

Supplementary Information supplementary informations

## Figures and Tables

**Table 1:** Analysis of variance (mean squares) of the morpho-physiological and qualitative traits for the accessions reported in Table S1, evaluated in four environments

Source	df	HD (days from 01/04)	PH (cm)	Sp/S (n°)	Se/S (n°)	TKW (g)	Ha (index)	PC (% d.b.)	SSV (mL)
Group (G)	8	998.07**	33692.16**	3.07	1691.37**	776.68**	7269.29**	109.69**	909.00**
Line (Group) [L(G)]	148	32.40**	357.37**	5.80**	103.70**	71.62**	701.00**	3.98**	210.82**
G × E	24	31.14**	1248.46**	5.61**	253.15**	25.88**	291.73**	5.05**	71.40**
[L(G)] × E	444	4.27**	49.24**	1.82	58.15**	9.58**	33.58**	1.19**	26.84**

G variance was tested using G × E variance, [L(G)] variance was tested using [L(G)] × E variance, G × E and [L(G)] variances were tested using the pooled error of the check cultivar Salmone. Level of statistical significance: * P < 0.05; ** P < 0.01.

df, degrees of freedom; HD, heading date; PH, plant height; Sp/S, number of spikelets per spike; Se/S, number of seeds per spike; TKW, thousand-kernel weight; Ha, hardness; PC, protein content; SSV, SDS sedimentation volume.
