# Supplementary material for: Diversity trends in bread wheat in Italy during the 20th century assessed by traditional and multivariate approaches
Source: Sci Rep. 2015 Feb 25;5:8574. doi: 10.1038/srep08574 (PMC4339800; doi:10.1038/srep08574)
Supplement: Supplementary Information — s [file srep08574-s1.pdf]

Diversity trends in bread wheat in Italy during the 20th century assessed by traditional and multivariate approaches

Leonardo Ormoli

Corrado Costa

Stefano Negri

Maurizio Perenzin

Patrizia Vaccino

Table S1. List of the materials, pedigree, year of release or registration and collection codes

Accession	Pedigree	Year[a]	SCV code[b]
Group 1 (Local landraces)			
Andriolo	unknown	n.a.	SCV-TA00040
Bianchetta	unknown	n.a.	SCV-TA00132
Bianco nostrale	unknown	n.a.	SCV-TA00141
Carosella	unknown	n.a.	SCV-TA00192
Carosella b	unknown	n.a.	SCV-TA00193
Chianti	unknown	n.a.	SCV-TA00210
Cologna lunga	unknown	n.a.	SCV-TA00230
Cologna lunga	unknown	n.a.	SCV-TA00231
Dente di cane	unknown	n.a.	SCV-TA00289
Gentilbianco	unknown	n.a.	SCV-TA00440
Gentilrosso	unknown	n.a.	SCV-TA00443
Gentilrosso	unknown	n.a.	SCV-TA00444
Gentilrosso	unknown	n.a.	SCV-TA00445
Gentilrosso	unknown	n.a.	SCV-TA00446
Gentilrosso	unknown	n.a.	SCV-TA00447
Majorca	unknown	n.a.	SCV-TA00657
Martinella	unknown	n.a.	SCV-TA00676
Rieti/1	unknown	n.a.	SCV-TA00881
Rieti/2	unknown	n.a.	SCV-TA00882
Rieti/3	unknown	n.a.	SCV-TA00883
Rieti/4	unknown	n.a.	SCV-TA00884
Rieti/5	unknown	n.a.	SCV-TA00885
Rieti/6	unknown	n.a.	SCV-TA00886
Rieti/7	unknown	n.a.	SCV-TA00887
Rieti/9	unknown	n.a.	SCV-TA00888
Rieti/10	unknown	n.a.	SCV-TA00889
Rieti/11	unknown	n.a.	SCV-TA00890
Solina	unknown	n.a.	SCV-TA01025
Solina	unknown	n.a.	SCV-TA01027
Solina 2	unknown	n.a.	SCV-TA01028
Solina 3	unknown	n.a.	SCV-TA01029
Solina 4	unknown	n.a.	SCV-TA01030
Solina 5	unknown	n.a.	SCV-TA01031
Solina 6	unknown	n.a.	SCV-TA01032
Solina a	unknown	n.a.	SCV-TA01033
Solina b	unknown	n.a.	SCV-TA01034
Group 2 (Selections from landraces)			
Caruso Girolamo (Inallettabile 8)	Selection from Inallettabile	n.a.	SCV-TA00533
Cologna 188	Selection from Cologna	n.a.	SCV-TA00225
Cologna 21 a	Selection from Cologna	n.a.	SCV-TA00226
Cologna 21 b	Selection from Cologna	n.a.	SCV-TA00227
Cologna 80	Selection from Cologna	n.a.	SCV-TA00228
Cologna 83	Selection from Cologna	n.a.	SCV-TA00229
Frassineto	Selection from Gentilrosso	n.a.	SCV-TA00397
Frassineto	Selection from Gentilrosso	n.a.	SCV-TA00398
Frassineto 405	Selection from Gentilrosso	n.a.	SCV-TA00399
Gentilrosso 13	Selection from Gentilrosso	n.a.	SCV-TA00448
Gentilrosso 160	Selection from Gentilrosso	n.a.	SCV-TA00449
Gentilrosso 4	Selection from Gentilrosso	n.a.	SCV-TA00450
Gentilrosso 48	Selection from Gentilrosso	n.a.	SCV-TA00452
Gentilrosso 58	Selection from Gentilrosso	n.a.	SCV-TA00454
Gentilrosso fam. 48	Selection from Gentilrosso	n.a.	SCV-TA00455
Gua' 113	Selection from Rieti	n.a.	SCV-TA00493
Inallettabile 8 (Girolamo Caruso)	Selection from Inallettabile	n.a.	SCV-TA00534
Inallettabile	Selection from Hatif Inversable Vilmorin	n.a.	SCV-TA00535

Inallettabile 96	Selection from Inallettabile	n.a.	SCV-TA00536
Inallettabile 210	Selection from Inallettabile	n.a.	SCV-TA00537
Inallettabile 3 (Vittorio Niccoli)	Selection from Inallettabile	n.a.	SCV-TA00538
Inallettabile 95	Selection from Inallettabile	n.a.	SCV-TA00539
Inallettabile 961	Selection from Inallettabile	n.a.	SCV-TA00540
Inallettabile Todaro	Selection from Inallettabile	n.a.	SCV-TA00541
Majorca 47	Selection from Majorca Bianca	n.a.	SCV-TA00658
Majorca 68	Selection from Majorca Bianca	n.a.	SCV-TA00659
Majorica 47	Selection from Majorca Bianca	n.a.	SCV-TA00660
Group 3			
Albimonte	Hatif Inversable Vilmorin/Akakomugi	1923	SCV-TA00028
Albimonte/b1	Hatif Inversable Vilmorin/Akakomugi	1923	SCV-TA00029
Albimonte/b2	Hatif Inversable Vilmorin/Akakomugi	1923	SCV-TA00030
Ardito	Wilhelmina Tarwe/Rieti 21//Akakomugi	1916	SCV-TA00057
Ardito/1	Wilhelmina Tarwe/Rieti 21//Akakomugi	1916	SCV-TA00058
Ardito/2	Wilhelmina Tarwe/Rieti 21//Akakomugi	1916	SCV-TA00059
Ardito/3	Wilhelmina Tarwe/Rieti 21//Akakomugi	1916	SCV-TA00060
Ardito/4	Wilhelmina Tarwe/Rieti 21//Akakomugi	1916	SCV-TA00061
Cambio	Rieti/Prince Albert	1920	SCV-TA00175
Catria	Hatif Inversable Vilmorin/Rieti	1923	SCV-TA00198
Mentana	Wilhelmina Tarwe/Rieti 21//Akakomugi	1918	SCV-TA00701
Villa Glori/1	Wilhelmina Tarwe/Rieti 67//Akakomugi	1918	SCV-TA01200
Villa Glori/2	Wilhelmina Tarwe/Rieti 67//Akakomugi	1918	SCV-TA01201
Villa Glori/3	Wilhelmina Tarwe/Rieti 67//Akakomugi	1918	SCV-TA01202
Group 4			
Apulia Precoce/1	Apulia/Ardito	1926	SCV-TA00048
Apulia Precoce/2	Apulia/Ardito	1926	SCV-TA00049
Aquila	S.Giovanni/Damiano	1936	SCV-TA00052
Ausonia	Wilhelmina Tarwe/Rieti 67//Akakomugi/3/Ardito	1928	SCV-TA00093
Autonomia	Frassineto 405/Mentana	1930	SCV-TA00094
Autonomia	Frassineto 405/Mentana	1930	SCV-TA00095
Autonomia A	Frassineto 405/Mentana	1938	SCV-TA00096
Autonomia B	Frassineto 405/Mentana	1938	SCV-TA00097
Bassi Lauro	n.a.	n.a.	SCV-TA00119
Comandante Baudi/1	Hatif Inversable Vilmorin/Rieti//Ardito	1926	SCV-TA00239
Comandante Baudi/2	Hatif Inversable Vilmorin/Rieti//Ardito	1926	SCV-TA00240
Impeto	Frassineto 405/Villa Glori	1930	SCV-TA00528
Libero (Littorio)	Apulia/Ardito	1927	SCV-TA00596
Libero (Littorio)/1	Apulia/Ardito	1927	SCV-TA00598
Libero (Littorio)/2	Apulia/Ardito	1927	SCV-TA00599
Libero (Littorio)/3	Apulia/Ardito	1927	SCV-TA00600
S. Pastore	Balilla/Villa Glori	1931	SCV-TA00946
S. Pastore/1	Balilla/Villa Glori	1931	SCV-TA00947
S. Pastore/2	Balilla/Villa Glori	1931	SCV-TA00948
Salto/1	Wilhelmina Tarwe/Rieti 67//Akakomugi/3/Ardito	1931	SCV-TA00970
Salto/2	Wilhelmina Tarwe/Rieti 67//Akakomugi/3/Ardito	1931	SCV-TA00971
Group 5			
Fortunato	Bassi Lauro /Damiano	1948	SCV-TA00391
Glutinoso	Ardito//Mottin/Norin 2	1957	SCV-TA00474
Leonardo	Bandi//S. Pastore/Damiano	1955	SCV-TA00586
Leone	S. Pastore/Funo-222-51-2//Carme 53/43	1955	SCV-TA00588
Mara/a	Autonomia/Aquila	1947	SCV-TA00662
Mara/b	Autonomia/Aquila	1947	SCV-TA00663
Mara/c	Autonomia/Aquila	1947	SCV-TA00664
Produttore S. 6	Salto//Sajtama-27/Quaderna	1954	SCV-TA00831
Group 6			
Aquileja	Tevere/Giuliari//Gallini	1974	SCV-TA00055
Argelato	Mara/Orlandi	1959	SCV-TA00064
Costante	Mara/Fortunato	n.a.	SCV-TA00256
Dardo	Mara/Fortunato	1963	SCV-TA00278
Dardo	Mara/Fortunato	1963	SCV-TA00279

Dardo	Mara/Fortunato	1963	SCV-TA00280
Frassino	Fortunato/Mara	1964	SCV-TA00400
Impetuoso	Impeto/Mara	n.a.	SCV-TA00531
Irnerio	Produttore S.6/Manitoba-2-P	1970	SCV-TA00545
Marzotto	Mara/Impeto	1959	SCV-TA00679
Libellula	Tevere/Giuliari//S. Pastore	1965	SCV-TA00594
Libellula	Tevere/Giuliari//S. Pastore	1965	SCV-TA00595
Lontra	Fortunato/Freccia//ATM 43	1963	SCV-TA00627
Orso	Funo/Produttore	1972	SCV-TA00777
Strampelli Nazareno	Libero//S.Pastore/Jacometti 49	1970	SCV-TA01069
Strampelli Nazareno	Libero//S.Pastore/Jacometti 49	1970	SCV-TA01070
Group 7			
Bolero	Linea 2625-267/Talent	1987	SCV-TA00153
Centauro	Irnerio/Strampelli	1983	SCV-TA00200
Eridano	Irnerio/SuperX	1989	SCV-TA00322
Gemini	Autonomia//Autonomia/Aquila	1981	SCV-TA00431
Leopardo	Leone/Libellula//Irnerio	1981	SCV-TA00591
Manital	Marzotto/Mendos	1981	SCV-TA00661
Mec	Marzotto/Combine	1974	SCV-TA00696
Oderzo	Argo/Ben Hur//Lontra/Ben Hur	1985	SCV-TA00769
Pandas	Orso//Bezostaja/S1//Generoso 7/C.Marzotto	1983	SCV-TA00796
Salmone	Bezostaja-1/Glutinoso	1980	SCV-TA00966
Group 8			
Apache	Axial/NRPB 844233	1998	SCV-TA00044
Barra	Marzotto/Mutant of Anza//Inia	1990	SCV-TA00118
Belfiore	Chiarano//PSV509/Centauro	1999	SCV-TA00122
Bilancia	Centauro/Chiarano	1996	SCV-TA00142
Colfiorito	Irnerio/IS 237 T//Argelato	1995	SCV-TA00221
Freccia	Pandas/119	1997	SCV-TA00401
Golia	Manital/Orso	1989	SCV-TA00477
Mieti	Mec/Vinci	1992	SCV-TA00708
Sagittario	Adam/Z282	1995	SCV-TA00961
Salgemma	Centauro/Gemini	1997	SCV-TA00964
Serio	Multiple cross	1993	SCV-TA01004
Group 9			
A 416	Genio/Centauro//Genio	2003	SCV-TA00001
Antille	n.a.	2006	SCV-TA01245
Artico	Multiple cross	2001	SCV-TA00081
Aubusson	Tremie/91B294	2003	SCV-TA01250
Blasco	Oderzo/Barra	2002	SCV-TA00145
Bologna	H89092/H89136//Soissons	2002	SCV-TA01253
Bramante	Victo/Soissons	2003	SCV-TA00158
Colledoro	Irnerio/Sel. CY (F5)//Falcon	2005	SCV-TA00222
Exotic	Etecho/Vivant	2005	SCV-TA00336
Geronimo	Bolero/Mieti	2003	SCV-TA00464
Isengrain	Apollo/Soissons	1997	SCV-TA00549
Palesio	Pandas/Recital	2000	SCV-TA00793
Palladio	Pandas/Soissons	2003	SCV-TA00794
PR22R58	Victo/FVP0040//XXC31	2002	SCV-TA00816

[a] n.a.: not available

[b] The code refers to the germplasm collection at CRA-SCV

Table S2. High Molecular Weight glutenin subunits (HMW-GS) composition and quality score of the accessions under study

SCV code	Accession	HMW-GS			*Quality score* [a]
		Glu-A1	*Glu-B1*	*Glu-D1*	
Group 1					
SCV-TA00040	Andriolo	N	7+8	2+10*	n.a.
SCV-TA00132	Bianchetta	N	7+8	2+12	8
SCV-TA00141	Bianco nostrale	1	7+8	2+12	9
SCV-TA00192	Carosella	2*	7+8	2+12	11
SCV-TA00193	Carosella b	1	7	2+12	7
SCV-TA00210	Chianti	1	7	2+12	7
SCV-TA00230	Cologna lunga	1	22	2+12*	3
SCV-TA00231	Cologna lunga	1	22	2+12*	3
SCV-TA00289	Dente di cane	1	7/20/13+19	2+12/5+10	n.a.
SCV-TA00440	Gentilbianco	N	20	2+12	5
SCV-TA00443	Gentilrosso	2*	13+19	2+12	7
SCV-TA00444	Gentilrosso	2*	7	2+12	9
SCV-TA00445	Gentilrosso	2*	7	2+12	9
SCV-TA00446	Gentilrosso	1	7	2+12	5
SCV-TA00447	Gentilrosso	1/2*	7	2+12	n.a.
SCV-TA00657	Majorca	2*	20/14+15	2+12	n.a.
SCV-TA00676	Martinella	1	7+8	2+12	9
SCV-TA00881	Rieti/1	1	13+19	2+12	5
SCV-TA00882	Rieti/2	1	13+19	2+12	5
SCV-TA00883	Rieti/3	1	13+19	2+12	5
SCV-TA00884	Rieti/4	1	13+19/7	2+12	n.a.
SCV-TA00885	Rieti/5	1	7	2+12	7
SCV-TA00886	Rieti/6	1	18*	2+12*	3
SCV-TA00887	Rieti/7	1	18*	2+12*	3
SCV-TA00888	Rieti/9	1	18*	2+12*/5+10	n.a.
SCV-TA00889	Rieti/10	N/1	6+8	2+12	n.a.
SCV-TA00890	Rieti/11	N/1	6+8	2+12**	n.a.
SCV-TA01025	Solina	1	6	2+12**	3
SCV-TA01027	Solina	1/N	6+8/7+8/20	2+12/2+12*/4+12*	n.a.
SCV-TA01028	Solina 2	1/2*	6+8/7+8	2+12	n.a.
SCV-TA01029	Solina 3	1/2*	20/7+8	2+12*/3+12	n.a.
SCV-TA01030	Solina 4	2*	6+8/7+8/13+19	2+12	n.a.
SCV-TA01031	Solina 5	2*/N	7+8	2+12/3+12	n.a.
SCV-TA01032	Solina 6	1/N/2*	7+8/6+8/13+16	2+12/2+12*	n.a.
SCV-TA01033	Solina a	1/2*	6/6+8	2+12**	n.a.
SCV-TA01034	Solina b	1	6	2+12**	3
				**Average quality score**	**6,0**
Group 2					
SCV-TA00533	Caruso Girolamo (Inallettabile 8)	N	7+8	2+12	8
SCV-TA00225	Cologna 188	N/1	13+19	2+12/5+10	n.a.
SCV-TA00226	Cologna 21 a	N	13+19	5+10	8
SCV-TA00227	Cologna 21 b	1/N	7	2+12/5+10	n.a.
SCV-TA00228	Cologna 80	N	13+19	2+12	4
SCV-TA00229	Cologna 83	N	13+19	2+12	4
SCV-TA00397	Frassineto	1	7	2+12	7
SCV-TA00398	Frassineto	1	7	2+12	7
SCV-TA00399	Frassineto 405	1	7	2+12	7
SCV-TA00448	Gentilrosso 13	N	7+8	2+12	8
SCV-TA00449	Gentilrosso 160	N	20	2+12	5
SCV-TA00450	Gentilrosso 4	1	7	2+12	7
SCV-TA00452	Gentilrosso 48	N	18*	2+12	4
SCV-TA00454	Gentilrosso 58	2*	7	2+12	9
SCV-TA00455	Gentilrosso fam. 48	1	7	2+12	7
SCV-TA00493	Gua' 113	N	7+8	2+12	8
SCV-TA00534	Inallettabile 8 (Girolamo Caruso)	N	7+8	3+12	8
SCV-TA00535	Inallettabile	N	20	2+12	5

SCV-TA00536	Inallettabile 96	N	18*	2+12*	2
SCV-TA00537	Inallettabile 210	1	7	2+12	7
SCV-TA00538	Inallettabile 3 (Vittorio Nicoli)	N	7+8	2+12	4
SCV-TA00539	Inallettabile 95	N	6+8	2+12	5
SCV-TA00540	Inallettabile 961	N	20	2+12	5
SCV-TA00541	Inallettabile todaro	N	20/6+8	2+12	n.a.
SCV-TA00658	Majorca 47	N	20	2+12	5
SCV-TA00659	Majorca 68	N	20	2+12	5
SCV-TA00660	Majorica 47	N	20	2+12	5
				**Average quality score**	**6,0**
Group 3					
SCV-TA00028	Albimonte	N	7+8	2+12	8
SCV-TA00029	Albimonte/b1	1	20	2+12	6
SCV-TA00030	Albimonte/b2	N	7+8	2+12	8
SCV-TA00057	Ardito	N	18+9	2+12	4
SCV-TA00058	Ardito/1	N	7+8	2+12	8
SCV-TA00059	Ardito/2	N	18+9	2+12	4
SCV-TA00060	Ardito/3	N	18+9	2+12	4
SCV-TA00061	Ardito/4	N	18+9	2+12	4
SCV-TA00175	Cambio	1	6+8	3+12	6
SCV-TA00198	Catria	N	20	3+12	5
SCV-TA00701	Mentana	N	7+8	2+12	8
SCV-TA01200	Villa Glori/1	1	20	2+12	6
SCV-TA01201	Villa Glori/2	1	7+8	2+12	9
SCV-TA01202	Villa Glori/3	1	7+8	2+12	9
				**Average quality score**	**6,4**
Group 4					
SCV-TA00048	Apulia Precoce/1	1	7+8	2+12	9
SCV-TA00049	Apulia Precoce/2	N	20	2+12	5
SCV-TA00052	Aquila	1	7+8	2+12	9
SCV-TA00093	Ausonia	N	20	2+12	5
CSV-TA00094	Autonomia	N	7+8	2+12	9
SCV-TA00095	Autonomia	1	7+8	2+12	9
SCV-TA00096	Autonomia A	2*	7+9	2+12	12
SCV-TA00097	Autonomia B	1	7+8	2+12	9
SCV-TA00119	Bassi Lauro	N	7+8	2+12	8
SCV-TA00239	Comandante Baudi/1	N	20	2+12	5
SCV-TA00240	Comandante Baudi/2	N	20	2+12	5
SCV-TA00528	Impeto	1	7+8	2+12	9
SCV-TA00596	Libero (Littorio)	N	6+8	2+12	5
SCV-TA00598	Libero (Littorio)/1	1	6+8	2+12	6
SCV-TA00599	Libero (Littorio)/2	1	26+27	2+12	5
SCV-TA00600	Libero (Littorio)/3	N	6+8	2+12	5
SCV-TA00946	S. Pastore	1	20	2+12	6
SCV-TA00947	S. Pastore/1	1	20	2+12	6
SCV-TA00948	S. Pastore/2	1	20	2+12	6
SCV-TA00970	Salto/1	N	20	2+12	5
SCV-TA00971	Salto/2	N	7+8	2+12	8
				**Average quality score**	**6,9**
Group 5					
SCV-TA00391	Fortunato	1	7+8	2+12	9
SCV-TA00474	Glutinoso	1	18*	2+12	5
SCV-TA00586	Leonardo	1	20	2+12	6
SCV-TA00588	Leone	1	7+8	2+12	9
SCV-TA00662	Mara/a	2*	17+18	2+12	13
SCV-TA00663	Mara/b	N	7	2+12	6
SCV-TA00664	Mara/c	N	7	2+12	6
SCV-TA00831	Produttore S. 6	N	7+8	2+12	8
				**Average quality score**	**7,8**
Group 6					
SCV-TA00055	Aquileja	1	7	2+12	7

SCV-TA00064	Argelato	N	7	2+12	6
SCV-TA00256	Costante	N	7	2+12	6
SCV-TA00278	Dardo	1	7/13+19	2+12	n.a.
SCV-TA00279	Dardo	2*	7+9	4+12	11
SCV-TA00280	Dardo	1	7/7+8	2+12	n.a.
SCV-TA00400	Frassino	N	7	2+12	6
SCV-TA00531	Impetuoso	1	7+8	2+12	9
SCV-TA00545	Irnerio	1	7+8	5+10	9
SCV-TA00679	Marzotto	1	20	2+12	6
SCV-TA00594	Libellula	1	20	2+12	6
SCV-TA00595	Libellula	1	20	2+12	6
SCV-TA00627	Lontra	1	7+8	2+12	5
SCV-TA00777	Orso	1	20	2+12	6
SCV-TA01069	Strampelli Nazareno	N	7+8	2+12	8
SCV-TA01070	Strampelli Nazareno	N	7+8	2+12	8
				**Average quality score**	**7,1**
Group 7					
SCV-TA00153	Bolero	2*	7+9	2+12	12
SCV-TA00200	Centauro	1	7+8	5+10	13
SCV-TA00322	Eridano	1	7+8	2+12	9
SCV-TA00431	Gemini	N	7	2+12	6
SCV-TA00591	Leopardo	1	7+8	5+10	13
SCV-TA00661	Manital	2*	17+18	2+12	13
SCV-TA00696	Mec	1	7	2+12	7
SCV-TA00769	Oderzo	1	7+8	5+10	13
SCV-TA00796	Pandas	1	7+9	2+12	10
SCV-TA00966	Salmone	1	7+9	2+12	10
				**Average quality score**	**10,6**
Group 8					
SCV-TA00044	Apache	N	7+9	2+12	9
SCV-TA00118	Barra	2*	7+9	5+10	16
SCV-TA00122	Belfiore	1	7+9	5+10	14
SCV-TA00142	Bilancia	1	7	5+10	11
SCV-TA00221	Colfiorito	2*	7+8	2+12	11
SCV-TA00401	Freccia	1	20	5+10	10
SCV-TA00477	Golia	1	17+18	2+12	11
SCV-TA00708	Mieti	1	7	2+12	7
SCV-TA00961	Sagittario	1	7+9	2+12	10
SCV-TA00964	Salgemma	1	7	2+12	7
SCV-TA01004	Serio	2*	7+9	5+10	16
				**Average quality score**	**11,1**
Group 9					
SCV-TA00001	A 416	N	7+8	2+12	8
SCV-TA01245	Antille	N	7+8	5+10	12
SCV-TA00081	Artico	N	7+8	2+12	8
SCV-TA01250	Aubusson	2*	7	5+10	13
SCV-TA00145	Blasco	2*	7+8	5+10	15
SCV-TA01253	Bologna	2*	7+8	5+10	15
SCV-TA00158	Bramante	2*	7+8	5+10	15
SCV-TA00222	Colledoro	N	7	2+12	6
SCV-TA00336	Exotic	N	6+8	5+10	9
SCV-TA00464	Geronimo	2*	7+8	5+10	15
SCV-TA00549	Isengrain	N	7+8	5+10	12
SCV-TA00793	Palesio	2*	6+8	2+12	8
SCV-TA00794	Palladio	1	7+9	5+10	14
SCV-TA00816	PR22R58	N	13+16	5+10	8
				**Average quality score**	**11,3**

[a] n.a.: not available

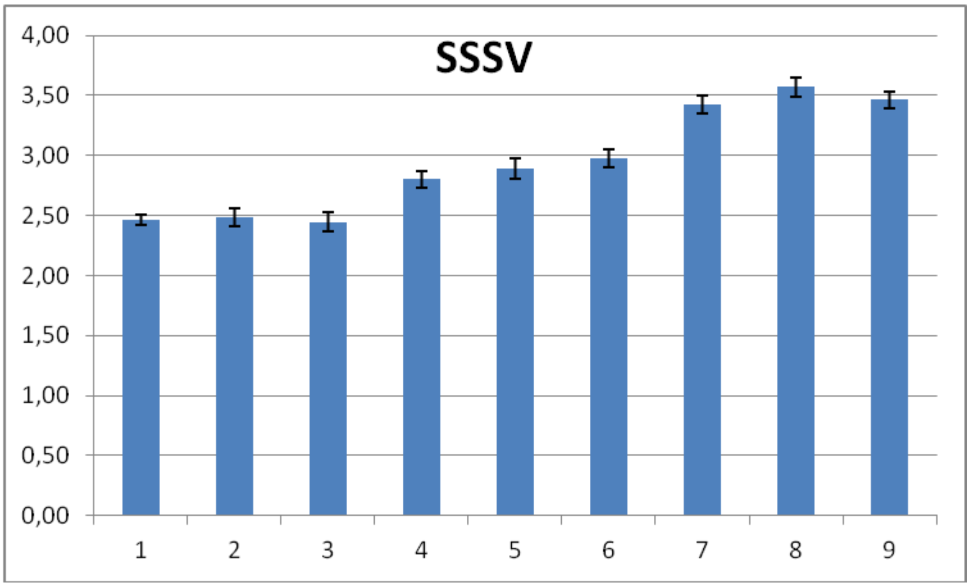

Fig. S1. Average specific SDS sedimentation volume (SSSV) of the nine cultivar groups across four environments. Error bars indicate standard errors; see supplementary Table S1for groups composition.
